# Vitamin A Enhances Macrophages Activity Against B16-F10 Malignant Melanocytes: A New Player for Cancer Immunotherapy?

**DOI:** 10.3390/medicina55090604

**Published:** 2019-09-18

**Authors:** Sofia Oliveira, José Costa, Isabel Faria, Susana G. Guerreiro, Rúben Fernandes

**Affiliations:** 1 School of Health, Porto Polytechnic (ESS, P. Porto), 4200 Porto, Portugal; sofiaramos030@gmail.com (S.O.); josetdacosta@gmail.com (J.C.); imf@ess.ipp.pt (I.F.); 2 Institute for Research and Innovation in Health (i3S), Porto University, 4200 Porto, Portugal; guerreiro.su@gmail.com; 3 Faculty of Medicine, University of Porto (FMUP), 4200 Porto, Portugal; 4 Faculty of Nutrition and Food Science, University of Porto (FCNAUP), 4200 Porto, Portugal

**Keywords:** melanoma, oxidative stress, vitamin A, redox modulation, immune response

## Abstract

*Background and objectives*: The incidence of cutaneous melanoma has been increasing. Melanoma is an aggressive form of skin cancer irresponsive to radiation and chemotherapy, rendering this cancer a disease with poor prognosis: In order to surpass some of the limitations addressed to melanoma treatment, alternatives like vitamins have been investigated. In the present study, we address this relationship and investigate the possible role of vitamin A. *Materials and Methods*: We perform a co-culture assay using a macrophage cell model and RAW 264.7 from mouse, and also a murine melanoma cell line B16-F10. Macrophages were stimulated with both *Escherichia coli* lipopolysaccharides (LPS) as control, and also with LPS plus vitamin A. *Results*: Using B16-F10 and RAW 264.7 cell lines, we were able to demonstrate that low concentrations of vitamin A increase cytotoxic activity of macrophages, whereas higher concentrations have the opposite effect. *Conclusion*: These findings can constitute a new point of view related to immunostimulation by nutrients, which may be considered one major preventive strategy by enhancing the natural defense system of the body.

## 1. Introduction

Skin cancers represent a major public health problem [1,2]. The incidence of cutaneous melanoma has increased over the years [3], and it can suppose a clinical and pathological challenge [4,5]. Cutaneous melanoma is a life-threatening skin tumor refractory to available therapies, like radiation and chemotherapy [5,6,7,8].

There are some established risk factors for melanoma development, like family history [9,10], genetic and hereditary factors, as well as environmental factors [1,2,3]. Nowadays, nutritional factors have emerged as possible modifiable risk factors for cutaneous melanoma, since a growing number of studies have shown that a balanced diet, rich in fruits and vegetables, is associated with lower risks for several types of cancer, including cutaneous melanoma. These nutrients have also been considered a possible alternative adjuvants to conventional therapies that are ineffective against this skin cancer type [1,3].

Vitamin A can be highlighted due to its role in normal and cancer conditions [11,12,13]. Vitamin A (retinol) is particularly important, since it cannot be synthesized by animals and, therefore, it must be supplied from the diet [1,14]. Vitamin A plays an critical role in cell differentiation [1,3], and has been demonstrated to inhibit several biological functions including tumor growth, angiogenesis and metastasis [14,15].

It could also be discussed if vitamin A active metabolites play some role in melanogenesis, but still this issue has not yet been elucidated [16,17]. The role of melanin pigment in melanoma biology has been known for several years [18,19]. Melanogenesis, the production of the melanin pigments in melanocytes, is being considered a molecular therapeutic target for melanoma [20]. Thus, targeting melanin production may control mutagenesis, promotion, and progression of melanoma [21,22,23], as well as enhancing the action of several therapeutic drugs, or even radiation therapy [24,25]. 

There are some evidences indicating the benefits of vitamin A in cancer therapy. However, the use of vitamin A for both chemoprevention or even for therapeutic applications remains controversial [1,14]. Accordingly, the main goal of the present study was to create and develop an in vitro model that could be used to address the use of antioxidant vitamins as therapeutic contributors capable of enhancing the immune system against cancer. In particular, we wanted to explore the redox effect of vitamin A in a melanoma cell line (B16-F10), as well as to understand its role in the immune system by assessing the activation of naïve macrophages (RAW 264.7). 

## 2. Materials and Methods

### 2.1. Cell Culture

The mouse melanoma B16-F10 cells exhibit in vitro a mixed morphology of spindle-shaped and epithelial-like cells. It was originally isolated from C57BL/6J mouse strain affected with melanoma. B16-F10 is a metastatic variant of B16 melanomas with high tropism for lung invasion.

The mouse RAW 264.7 cells exhibit in vitro a morphology of monocyte/macrophage cells and were originally isolated from Abelson murine leukemia virus-induced tumor of the BALB/c mouse strain.

B16-F10 murine melanoma cell line (ATCC CRL-6475) and RAW 264.7 macrophage-like cell line (ATCC TIB-71) were maintained in Dulbecco’s modified eagle’s medium (DMEM; Sigma-Aldrich, Saint Louis, MO, USA). The media formulation was supplemented with 10% heat-inactivated fetal bovine serum (FBS; Sigma-Aldrich) and 1% penicillin/streptomycin/amphotericin B mixture (Sigma-Aldrich). Cells were grown at 37 °C under a humidified atmosphere with 5% CO_2_. B16-F10 (10^4^ cells/mL) and RAW 264.7 (2 × 10^4^ cells/mL) were seeded in 96-wells plates and allowed to adhere for 24 h. Unless otherwise specified, all treatments and controls were carried out in serum-free conditions [26].

### 2.2. Dose–Response Assay to H_2_O_2_-Induced Oxidative Stress

To determine the accurate hydrogen peroxide (H_2_O_2_) concentration to induce oxidative stress [27,28], B16-F10 malignant melanocytes were treated with different H_2_O_2_ concentrations (10, 25, 50, 75, and 100 µM) for 24 h. The experimental concentration was determined by thiobarbituric acid reactive substances (TBARS) assay, and cell metabolic activity was measured by 3-(4,5-dimethylthiazol-2-yl)-2,5-diphenyltetrazolium bromide (MTT) assay. Cells incubated with serum-free medium were considered the controls to this experiment (results not shown).

### 2.3. TBARS Assay

B16-F10 cells (10^5^ cells/mL) were seeded in 24-wells plates and allowed to adhere for 24 h. Afterwards, melanoma cells were cultured with standard H_2_O_2_ treatments in serum-free conditions for 24 h. Then 50% trichloroacetic acid (TCA; Sigma-Aldrich) was added to each treatment. The mixture was centrifuged (6000 rpm, 2 min, 4 °C) and 1% thiobarbituric acid (TBA; Sigma-Aldrich) was added to the supernatant that was maintained in the water-bath for 40 min. The detection was performed using the Jenway 6405 UV/Vis spectrophotometer (Felsted, UK) at wavelength absorbance of 535 nm (results not shown).

### 2.4. Dose–Response Assay to Vitamin A

To determine the accurate vitamin A concentration responsible for significant alterations in cell metabolic activity, B16-F10 malignant melanocytes were incubated with different vitamin A concentrations (0.1, 1.0, 10.0, and 100.0 µg/mL) for 24 h. Cell metabolic activity was measured by MTT assay and the experimental concentrations were defined as the lowest value that does not considerably alter the cell viability, and the highest value that considerably alters the cell viability. Cells incubated with serum-free medium were considered the controls for this experiment (results not shown).

### 2.5. Combined Effects of Vitamin A and H_2_O_2_

To determine the possible redox effect of vitamin A, B16-F10 malignant melanocytes were previously submitted to a H_2_O_2_ treatment (25 µM) for 24 h. Then B16-F10 cells were submitted to two different concentrations of vitamin A (0.1 and 100.0 µg/mL) for 24 h. Cell metabolic activity was measured by MTT assay. Cells incubated with serum-free medium were considered the controls for this experiment. 

### 2.6. Metabolic Activity Determination: MTT Assay

To measure B16-F10 and RAW 264.7 cell activity, after a 24 h incubation with different treatments, 20 µL of the 3-(4,5-dimethylthiazol-2-yl)-2,5-diphenyltetrazolium (MTT) (Sigma–Aldrich) was added into each 96-plate well, followed by a 3 h incubation period. Assays using tetrazolium salts are generally used to evaluate proliferation and cell viability. Cell proliferation is defined as the cellular growth rate and cell viability determined by the number of live cells, and is usually expressed as a percentage of the control [29]. The determination of live cells is generally performed by staining them with a vital dye under the hemocytometer where it is calculated the number of stained cells (alive) by the total (stained plus non-stained, or dead). Tetrazolium salts are commonly used as cell viability measuring methods, which is maybe true, but the accuracy of the expression may be discussed. Tetrazolium salts are cleaved to formazan by the succinate–tetrazolium reductase system (EC 1.3.99.1) which belongs to the respiratory chain of the mitochondria, and is only active in metabolically intact cells. In the present work, we will define the increased reduction of tetrazolium salts to a purple dye as metabolic activity. Color development was determined by measuring absorbance at 550 nm by Thermo Electrocorporation Multiskan Ascent^®^ (Shangai, China) microplate reader (data were obtained from Ascent Software for Multiscan Ascent^®^, version 2.6) [30].

### 2.7. Macrophage-Mediated Cytotoxicity

RAW 264.7 macrophage-like cells were incubated with lipopolyssacharides (LPS) from *Escherichia coli* O111:B4 (LPS L4391, Sigma–Aldrich) 1000 ng/mL. The activated macrophages were co-incubated with B16-F10 melanoma cells (10:1 ratio) at 37 °C in a 5% CO_2_ incubator for 24 h. Then cells were treated with different concentrations of vitamin A (0.1 and 100.0 µg/mL), and the cytotoxic effects of LPS-activated macrophages–melanoma cells were measured, calculated, and expressed as follows, were OD stands for optic density for the MTT assay [31]:

% Cytotoxicity = [1 − (OD of (melanoma cells + macrophages) − OD of macrophages)/(OD of melanoma cells (untreated))] × 100

### 2.8. Statistical Analysis

The variables were expressed as mean ± SD (standard deviation). Data were analyzed with GraphPad Prism 6.0 (GraphPad Software Inc., San Diego, CA, USA). When three or more conditions were evaluated, statistical analysis was conducted through one-way ANOVA with Sidak post hoc test for comparisons between control and treatments (H_2_O_2_ and vitamin A treatments), and with Tukey’s multiple comparisons test for comparisons between every different treatment/condition. Significance was set at *p* < 0.05.

## 3. Results

### 3.1. Lower Concentrations of Vitamin A May Be Related to Its Possible Antioxidant Properties

**Figure 1:**
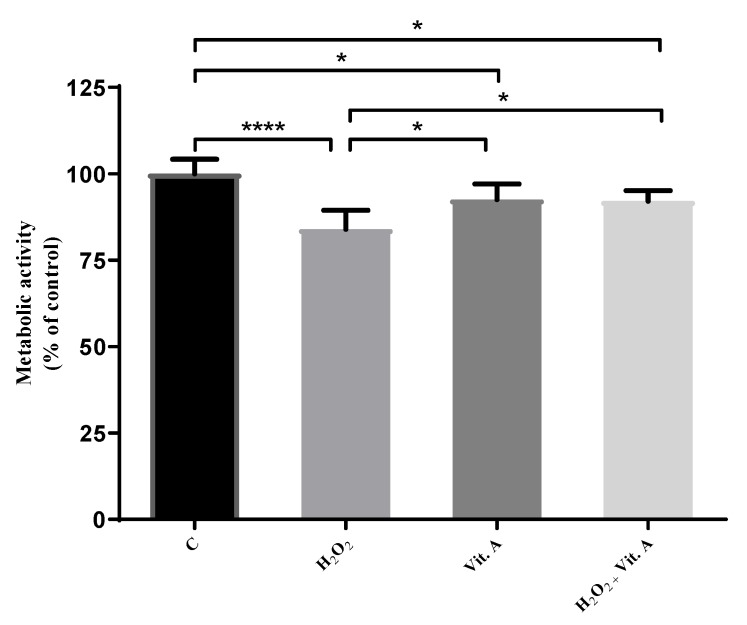
The effect of vitamin A (0.1 µg/mL) in redox modulation on B16-F10 melanoma cells by MTT assay. Control (C), H_2_O_2_ (25 µM treatment). After 24 h, (0.1 µg/mL) vitamin A was used. C represents cells incubated with serum-free medium. Results of metabolic activity of B16-F10 cells represent the percentage (%) of treated cells normalized over the absorbance of control, and were represented as mean ± SD. The results were statistically significant when *p* < 0.05. H_2_O_2_ decreased the metabolic activity of B16-F10 cells when compared to control (*p* < 0.0001). H_2_O_2_ + vitamin A revert the decrease of metabolic activity when compared to H_2_O_2_ treated cells (*p* < 0.05). * *p* < 0.05; **** *p* < 0.0001.

After the establishment of a dose versus response curve for the assessment of vitamin A effects (results not shown), the possible redox effects of the concentration of 0.1 µg/mL were studied. These results are shown in Figure 1.

Figure 1 compares the cell metabolic activity of the control group to H_2_O_2_ treated cells, as well as vitamin A treated cells and H_2_O_2_ + vitamin A treated cells. Metabolic activity of cells allowed us to study viability of the cells on different conditions of treatments. All of these comparisons are significant. H_2_O_2_ treatments significantly reduce the metabolic activity of melanoma cells (*p* < 0.0001). The same evidence is found in vitamin A treatment (*p* < 0.05), and in the combined treatment of H_2_O_2_ + vitamin A (*p* < 0.05).

Figure 1 also compares the action of vitamin A in cells treated under oxidative stress caused by H_2_O_2_. Results demonstrate that 0.1 µg/mL vitamin A may buffer the effect caused by H_2_O_2_ (*p* < 0.05), since there is no significant difference between cells treated with vitamin A alone and those treated with the vitamin and the same amount of H_2_O_2_ (25 µM) that significantly decreases metabolic activity. 

### 3.2. Higher Concentrations of Vitamin A May Be Related to Its Possible Pro-Oxidant Properties

**Figure 2:**
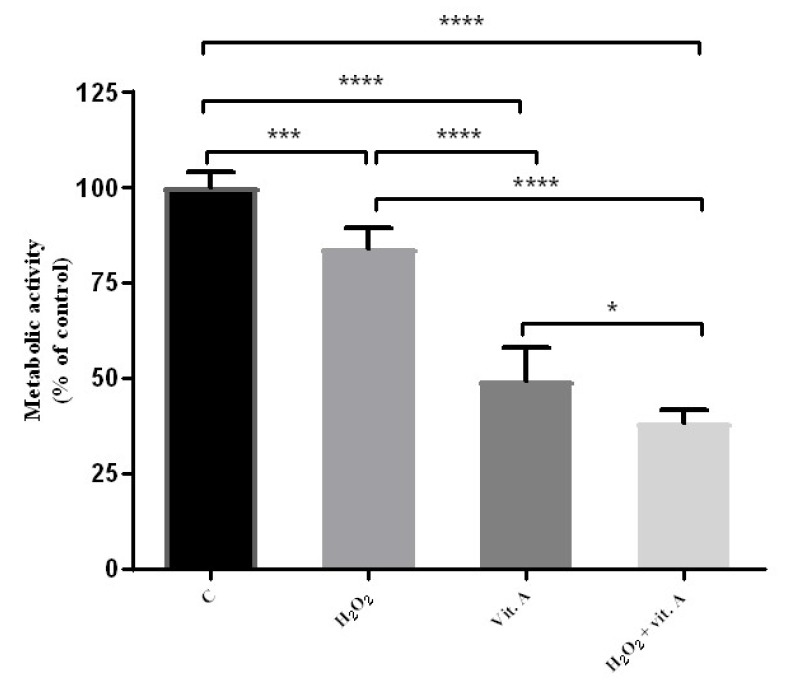
The redox effect of vitamin A (100 µg/mL) on metabolic activity of B16-F10 melanoma cells. H_2_O_2_ (25 µM treatment) and vitamin A treatments alone are capable of reducing cell metabolic activity. However, treatment combination (H_2_O_2_ + vitamin A) has a stronger effect on reducing metabolic activity, which may suggest a possible pro-antioxidant role of vitamin A (100 µg/mL). C represents cells incubated with serum-free medium. Results represent the percentage (%) of treated cells normalized over the absorbance of control. and were represented as mean ± SD. * *p* < 0.05, *** *p* < 0.001; **** *p* < 0.0001.

The possible redox effects of the concentration of 100 µg/mL were also studied. These results are shown in Figure 2.

Figure 2 compares cell metabolic activity of the control group to H_2_O_2_, to vitamin A, and to H_2_O_2_ + vitamin A treated cells. All of these comparisons are significant. H_2_O_2_ (25 µM) treatments significantly reduce the metabolic activity of melanoma cells (*p* < 0.0001), as well as 100 µg/mL vitamin A (*p* < 0.0001) and H_2_O_2_ + 100 µg/mL vitamin A treatments (*p* < 0.0001). Figure 2 also compares H_2_O_2_ treated cells to vitamin A treated cells and to H_2_O_2_ + vitamin A treated cells. All the comparisons are significant.

When comparing H_2_O_2_ treated cells to cells treated with 100 µg/mL vitamin A, it is possible to observe an even more significant decrease (*p* < 0.0001) in the metabolic activity of the tumor cells. Additionally, comparing the effect of 100 µg/mL vitamin A treatment in cells previously submitted to 25 µM H_2_O_2_, not only the buffering effect of 0.1 µg/mL vitamin A was not observed. Contrariwise, an even lower metabolic activity was found (*p* < 0.0001) in melanoma cells than the metabolic activity achieved with both treatments (25 µM H_2_O_2_ and 100 µg/mL vitamin A) alone. 

### 3.3. LPS Is Capable of the Activation of RAW 264.7 Naïve Cells

**Figure 3:**
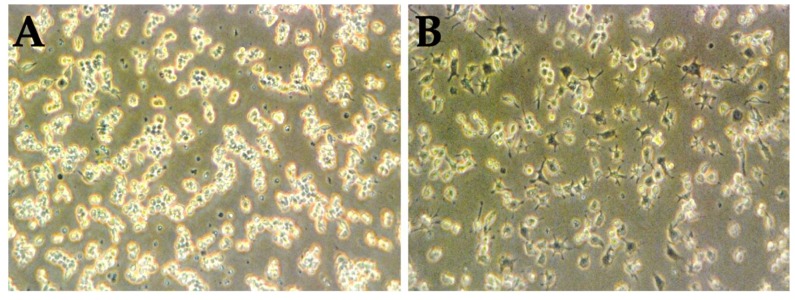
RAW 264.7 macrophage-cells like before (**A**) and after (**B**) LPS treatment (200×).

For the assessment of macrophage cytotoxicity, RAW 264.7 cells were previously incubated with 1 µg/mL LPS. After 24 h, cells were also incubated with 0.1 and 100.0 µg/mL vitamin A concentrations. Figure 3 shows the phenotype of RAW 264.7 cells before LPS treatment (Figure 3A), and after LPS treatment (Figure 3B). 

Figure 3 shows the influence of LPS treatment on RAW 264.7 macrophage-like cells phenotype. Macrophage-like cells without LPS activation presents a round shape morphology (Figure 3A), and cells with LPS activation presents a macrophage-like shape with some pseudopods (Figure 3B).

### 3.4. The Percentage of Macrophage-Mediated Cells Cytotoxicity Is Inversely Correlated to Cell Culture Results Alone

After LPS activation, two groups were treated with the two different concentrations of vitamin A previously used. According to the cytotoxic algorithm created by Seo and collaborators [31], the percentage of cytotoxicity mediated by macrophages was assessed in the conditions defined for the present experiment. The results of this experiment are shown in Table 1.

## 4. Discussion

Despite all the knowledge about melanoma biology, this cancer remains one of the greatest challenges of our day [1,5,32,33], mainly due to the acquired resistance to chemotherapy and radiation [5,30]. Therefore, melanoma treatment remains essentially surgical [1,6,7,10].

Although the diagnosis is relatively easy, there is an emerging need for new compounds capable of preventive or curative properties. That way, there are growing fields that state the possible role of dietary factors [1,11], as well as the immunostimulation by external compounds [31].

Emerging evidence indicates that dietary factors, such as antioxidant compounds, could have a double role, acting as an antioxidant and pro-oxidant, depending on the concentrations used [34,35,36]. Some studies suggest that these compounds could be used in cancer prevention and/or treatment [37,38,39].

According to this line of evidence, Figure 1 and Figure 2 show that H_2_O_2_ and vitamin A treatments alone are capable of reducing cell metabolic activity in a significant way. When treatments are combined, 0.1 µg/mL vitamin A has the capability of an increase in cell metabolic activity that surpasses the percentage of metabolic activity of H_2_O_2_ treatment alone (Figure 1). At this point, these data may suggest a possible antioxidant role of lower concentrations of vitamin A in melanoma cells. On the other hand, when treatments are combined, 100 µg/mL vitamin A has the capability of a decrease in metabolic activity that is lower than H_2_O_2_ treatment alone (Figure 2). Accordingly, these data may suggest a possible pro-oxidant role of higher concentrations of vitamin A. However, further studies are needed to prove this possibility.

These in vitro model experiments gave a pool of results that allows us to suggest that vitamin A could have a possible double role, acting as an antioxidant and pro-oxidant, depending on the vitamin A concentration used. In this manner, and in order to assess the effect of vitamin A in a more complex environment, more experiments were done. For that, an immune response induction model was used based on the work developed by Seo and collaborators [31].

The present model establishes a co-culture of both macrophages RAW 264.7 and B16-F10 melanoma cells. Under basal conditions, when macrophages are activated with LPS, they are able to limit tumor cells activity in a basal cytotoxic level of 73.8% (Table 1). This value is considered the baseline for this experiment and every group previously submitted to LPS activation.

After LPS activation, two groups of macrophages were treated with different amounts of vitamin A. Macrophages stimulated with 0.1 µg/mL vitamin A achieved a cytotoxicity of 86.8% (13% higher), whereas 100 µg/mL vitamin A treatment presented a cytotoxicity of only 75.7%. Thus, activated macrophages stimulated with 100 µg/mL vitamin A treatment presented a slight increased cytotoxicity of only 1.9%.

These results show the capability of lower concentrations of vitamin A to increase the macrophage activity more effectively than higher concentrations of vitamin A treatment, thus enhancing macrophage-induced cytotoxicity.

If this work ended at the redox studies, one may suggest that vitamin A could have a possible antitumor effect/role [34,35,36]. However, the co-culture experiment allowed the study of different concentrations of vitamin A in a more complex environment. In this experiment, the possible therapeutic effect of vitamin A was inversely correlated to the results of the melanoma cells culture alone. According to these data, when the vitamin A dose is lower, its antitumor activity is enhanced in a more efficient way, since macrophage-melanoma cells binomial shows a decrease of tumor cells metabolic activity (Table 1).

## 5. Conclusions

The discussion of the effect of vitamins in the literature is almost inconclusive, since there are no studies devoted to the investigation of the dose–response effect of those vitamins. Although these results are encouraging, new questions are now evolving. Given that there is little information about vitamin A and melanoma, further studies should be done in order to validate the results presented in this study.

## Figures and Tables

**Table 1:** Percentage (%) of macrophage-mediated cells cytotoxicity.

Cells and Treatments	Cytotoxicity (%)
Control (without vitamin A)	73.8%
Vitamin A treatment [0.1 µg/mL]	86.8%
Vitamin A treatment [100 µg/mL]	75.7%
